# Tolerance to Gamma Radiation in the Tardigrade *Hypsibius dujardini* from Embryo to Adult Correlate Inversely with Cellular Proliferation

**DOI:** 10.1371/journal.pone.0133658

**Published:** 2015-07-24

**Authors:** Eliana Beltrán-Pardo, K. Ingemar Jönsson, Mats Harms-Ringdahl, Siamak Haghdoost, Andrzej Wojcik

**Affiliations:** 1 Department of Molecular Biosciences, The Wenner-Gren Institute, Stockholm University, Stockholm, Sweden; 2 School of Education and Environment, Kristianstad University, Kristianstad, Sweden; University of North Carolina, UNITED STATES

## Abstract

Tardigrades are highly tolerant to desiccation and ionizing radiation but the mechanisms of this tolerance are not well understood. In this paper, we report studies on dose responses of adults and eggs of the tardigrade *Hypsibius dujardini* exposed to gamma radiation. In adults the LD_50/48h_ for survival was estimated at ~ 4200 Gy, and doses higher than 100 Gy reduced both fertility and hatchability of laid eggs drastically. We also evaluated the effect of radiation (doses 50 Gy, 200 Gy, 500 Gy) on eggs in the early and late embryonic stage of development, and observed a reduced hatchability in the early stage, while no effect was found in the late stage of development. Survival of juveniles from irradiated eggs was highly affected by a 500 Gy dose, both in the early and the late stage. Juveniles hatched from eggs irradiated at 50 Gy and 200 Gy developed into adults and produced offspring, but their fertility was reduced compared to the controls. Finally we measured the effect of low temperature during irradiation at 4000 Gy and 4500 Gy on survival in adult tardigrades, and observed a slight delay in the expressed mortality when tardigrades were irradiated on ice. Since *H*. *dujardini* is a freshwater tardigrade with lower tolerance to desiccation compared to limno-terrestrial tardigrades, the high radiation tolerance in adults, similar to limno-terrestrial tardigrades, is unexpected and seems to challenge the idea that desiccation and radiation tolerance rely on the same molecular mechanisms. We suggest that the higher radiation tolerance in adults and late stage embryos of *H*. *dujardini* (and in other studied tardigrades) compared to early stage embryos may partly be due to limited mitotic activity, since tardigrades have a low degree of somatic cell division (eutely), and dividing cells are known to be more sensitive to radiation.

## Introduction

Tardigrades are small aquatic invertebrates known to tolerate a number of severe stressors, including very high and low temperatures, complete desiccation under vacuum and high doses of ionizing radiation [1]. Beginning with the seminal paper by May et al. [2] several studies on radiation tolerance of both adult tardigrades and their eggs have been published. These studies include both low-LET radiation (X-rays, [2]; gamma rays, [3,4]), high-LET radiation (alpha particles, [4]; protons, [5]), UV radiation [6,7], and combinations of cosmic and UV radiation under space conditions [8]. The results from these studies position tardigrades among the most radiation-tolerant animals, with LD50 values of adults within the first 2 days after irradiation in the range of 3 to 10 kGy for ionizing radiation. Several studies have also shown that tolerance to ionizing radiation is independent of whether the animals are irradiated in a hydrated or desiccated state [2,3,4], but Horikawa et al. [7] found that tolerance to UVC was higher when tardigrades were irradiated in a desiccated state. A few studies of radiation tolerance in tardigrade eggs have also been reported, and as expected eggs have a much lower tolerance compared to adults [9–11]. Previous studies have also documented differences in tolerance to radiation among different tardigrade species [7,8]. Most species studied belong to the ecological category of “limno-terrestrial” species inhabiting microenvironments that regularly dry out, and the tolerance to extreme unnatural stressors such as ionizing radiation is supposed to be a by-product of adaptations to survive in dry environmental conditions [12].

The only non-limno-terrestrial tardigrade species that have been used in radiation studies so far is the freshwater species *Hypsibius dujardini*. Horikawa et al. [7] used this species together with the limno-terrestrial species *Ramazzottius varieornatus* in a study on tolerance to UVC radiation and DNA damage/repair, and documented a much higher radiation tolerance in *R*. *varieornatus*. They also provided evidence of a lower tolerance to desiccation in *H*. *dujardini* (no survival under desiccation at 33.8% relative humidity, compared to 99% survival in *R*. *varieornatus*). Also Wright [13] found lower desiccation tolerance in *H*. *dujardini* compared to several other tardigrades that inhabit limno-terrestrial microhabitats. In view of the indicated lower tolerance to desiccation and UV radiation in *H*. *dujardini* we found it valuable to investigate if also tolerance to gamma radiation is lower in this species. *H*. *dujardini* represents an interesting tardigrade species for radiation studies also because it is one of the few tardigrades where the embryonic development has been studied in details [14]. Cellular activities during embryonic development (e.g., mitosis, regulation of genes/proteins) may provide important information for understanding the mechanisms of radiation tolerance, and in two previous studies we have shown that the tolerance to radiation in tardigrade embryos increases as they develop [10,11].

In the present paper, we report a study on radiation tolerance in adults and embryos at different developmental stages in *H*. *dujardini*. We also evaluate the effect of low temperature during irradiation on post-irradiation survival and reproduction.

## Methods

### Animals used in the study

Animals from a laboratory population of the eutardigrade *Hypsibius dujardini* were provided by the Biology Department of University of North Carolina at Chapel Hill (Prof. B. Goldstein). This population originates from a benthic sample at Darcy Lever, Bolton, Lancashire, England (British National Grid Reference SD741078) [14]. The population propagates through parthenogenesis, has a generation time of about two weeks, and an egg development time of 4–5 days [14]. Cultures were kept in distilled water in 9 cm petri dishes at an average temperature of 20 C°, and were fed by algae of *Chlorococcum* sp. Medium was changed every 10 days.

### Radiation source and measurement of radiation effects

Tardigrade samples were irradiated with gamma rays in a Gammacell 1000 ^137^Cs source (Isomedix, Inc., Kanata, Ontario, Canada) at a dose rate of 6.04 Gy/min. Effects of radiation on animal survival has previously been estimated by Jönsson et al. [3] from the viability criteria: (i) dead with extended body and no movement; (ii) alive but without coordinated leg movements; and (iii) alive with coordinated leg movements. In this study we did not distinguish between conditions (ii) and (iii) but included both within the category of viable animals. Animal fertility was estimated as the number of eggs laid during a certain period of time, while viability of eggs represent the proportion of eggs that hatched. All irradiations were made on active hydrated animals or eggs.

### Dose-response experiment with adult tardigrades

In order to evaluate the effect of gamma radiation on survival of adults, groups of 20 adults were selected and placed in 19mm diam. petri dishes with 1ml of distilled water and agar as a base for walking. Irradiation of the samples was performed at room temperature at the following doses: 0, 100, 500, 700, 900, 1000, 2000, 3000, 4000, 4500 and 5000 Gy. After irradiation the animals were transferred to 30 mm petri dishes with 2 ml of distilled water and 100 μl of algae culture (*Chlorococcum* sp.). Survival of animals along with their capacity to propagate (fertility, egg hatchability) was monitored daily until 12–20 days post-irradiation. To measure egg viability the eggs laid were transferred to new petri dishes with the same conditions (the algae was added only after the first hatch) in order to record hatching rate. The eggs were observed until hatched, ranging from 4 to 10 days post-laying.

### Dose-response experiment with eggs at different stages of development

The effect of gamma radiation on hatchability of *H*. *dujardini* eggs was evaluated according to a previously established protocol for *Milnesium tardigradum* [11], modified for time when the eggs were irradiated since total development time is different in the two species. As a reference for defining the two developmental stages used in our study, we used the study of Gabriel et al. [14]: Initial stage of development, 0 to 6 hours post-laying; Late stage of development, 72 to 77 hours post-laying. Pictures of embryos were taken to verify the reached developmental stage at these times of development. For dose-response analysis we used 3 doses plus control per developmental stage (0 Gy, 50 Gy, 200 Gy and 500 Gy) and 3 repeats per dose, with 15 eggs in each repeat. The eggs were monitored until 18 days post-laying. Within this period the eggs started to hatch and the juveniles were separated and fed with 30 μl of algae culture, allowing them to develop into young adults. The survival rate of these animals was measured over the interval of day 11–20 or 10–20 post-laying (for early and late dev. group, respectively), and was calculated as the number of young adults divided by the number of hatched eggs. The days 10 or 11 were selected as initial points of survival monitoring since these represented the last days when viable irradiated eggs hatched. The fertility, based on number of eggs laid, was measured, and relative fertility calculated as mean number of eggs laid (in each replicate sample) divided by the highest number of young adults available per repeat. Eggs laid within each replicate sample were removed continuously.

Additionally, 10 eggs were irradiated in the initial stage of development with 50 Gy and 6 were also selected as controls. These eggs were photographed (with phase contrast in a Nikon Eclipse E600 microscope) before and after irradiation, and every day during their development until hatch in order to observe changes caused by radiation. The following developmental times and descriptions from the study of Gabriel et al. [14] were used as reference to establish the morphological changes observed in the embryos: stage 2, two cell (2 hours post laying (hpl)); stage 3, four cell (∼ 3 hpl); stages 4–6, 8 to 32 cells (∼ 4–6.5 hpl); stages 7–10, 60 to ∼ 500 cells (∼ 7.5–16.5 hpl); stage 11, elongation and comma shape of the whole embryo (∼ 17 hpl); stages 12–13, appearance of segmental units (∼ 21 hpl); stage 14, limb bud formation (∼ 26 hpl), with no detection of cell division in this region at this time; stage 15, pharynx and buccal apparatus can be seen (∼ 28.5–38.5 hpl); stage 16, ganglia and midgut visible (∼ 38–50 hpl); stage 17, muscle twitching, presence of stylets, claws, and eyespots. The embryo rotates inside the egg shell (∼ 40–50 hpl); stage 18, pharynx and buccal apparatus morphologically distinct (∼ 52–65 hpl); stage 19, hatching. The embryos hatched 4 to 4.5 days after being laid.

### Effect of low temperature during irradiation on adult survival and fertility

In this experiment we evaluated if there was an effect on survival of adults when irradiation was performed at different temperatures. Animals with no visible eggs in the body were selected. Two treatment groups were used, *irradiation on ice* and *irradiation without ice* (= room temperature), and the experiment included 4 doses (100 Gy, 200 Gy, 4000 Gy and 4500 Gy) plus control. These doses were chosen based on the results from the dose response experiment with adult tardigrades, which showed that only doses above 3000 Gy affected survival and that effects on fertility and hatchability were observed in the interval between 100 and 500 Gy (see Results section). For each dose we used 3 repeats with 15 animals in each. Before the irradiation, animals selected for irradiation on ice were kept at 4°C for 15 hours to avoid stress. A metal container with a water mantle was frozen to -80°C, filled with ice and placed in the irradiation chamber. Thirty mm petri dishes with animals were placed on ice and also covered with ice (0–0.2°C). The “without ice” treatment group was irradiated at ambient room temperature. The control sample (0 Gy) was left next to the irradiation source in a similar metal container. During irradiation no food or water was added but the ice was replaced after 7 hours of irradiation to maintain the low temperature. Immediately after irradiation the animals were counted, 500 μl of water and 50 μl of algae was added, and then all animals were kept at room temperature. Survival data of animals was collected after 12 hours and then once per day for 12 days, and were thereafter monitored for 22 days and 28 days in order to measure their fertility and the viability of eggs, respectively.

### Statistical analyses

We used nonparametric variance analyses (Kruskal-Wallis Analysis of Variance, H used for notation of test statistics; Mann-Whitney U-test, U used for notation of test statistics) for statistical comparisons among treatment groups. Complete statistical results are reported in the Supporting Information File (Data in S1 Text).

## Results

### Effects of irradiation on adult survival and reproduction

**Fig 1:**
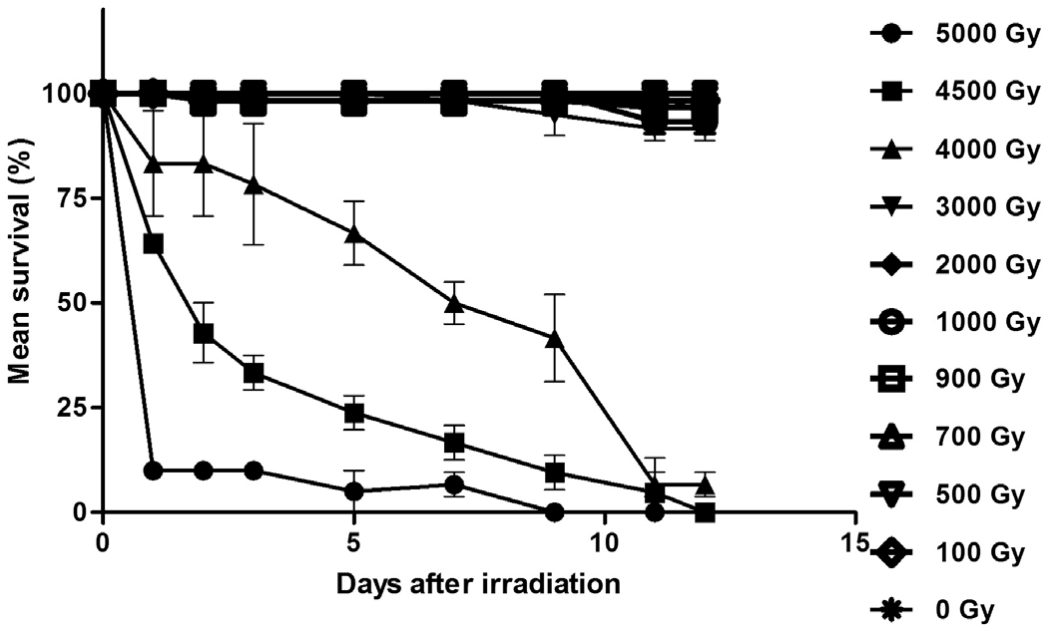
Survival of adult *H*. *dujardini* after exposure to different doses of gamma radiation. Error bars represent standard deviations from 3 repeats, each with 20 animals.

Doses of gamma ray up to 3000 Gy did not affect the survival of *H*. *dujardini* (No statistical difference between doses from 0 to 3000 Gy at any of the 1–12 time estimates; Kruskal-Wallace analysis, p>0.05 for all estimates; Fig 1 and S1 Text). Survival for all doses above 3000 Gy differed significantly from the control (p<0.05 at all estimates), and this was true at most time estimates also among the three high-dose groups (4000, 4500, 5000 Gy). The only exception was the 4500 and 5000 Gy groups that did not differ at day 11 (U = 6.0, p = 0.32) and 12 (U = 4.5, p = 1.00). A dose of 4000 Gy reduced the survival to 83% after 24 and 48 hours and survival then declined relatively linearly with time to 40% after 9 days (Fig 1). For doses of 4500 and 5000 Gy the initial decline in survival was more pronounced and reached 40% after 4500 Gy and 10% after 5000 Gy 24 hours post-irradiation. The reduction in survival over time after the initial 24h decline was much slower, particularly for the 5000 Gy group. The LD_50/48h_ was estimated to be 4180 Gy (Table Curve 2D 5.01 with Transition functions; Best fit: Log normal cumulative function, r^2^ = 0.9995).

**Fig 2:**
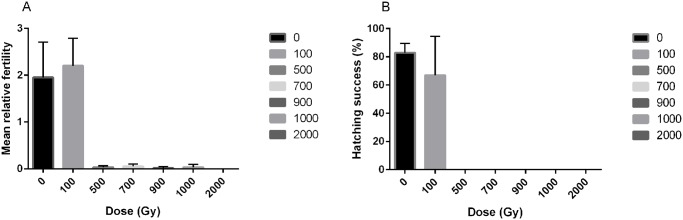
Radiation effects on fertility of the tardigrade *H*. *dujardini*. A) Fertility levels expressed as the average number of eggs laid during 12 days after irradiation. B) Egg viability expressed as the proportion of laid eggs that hatched. Error bars represent standard deviations from 3 repeats, each with 20 animals.

The effect of irradiation on fertility and egg viability in *H*. *dujardini* was also evaluated (Fig 2 and S1 Text). A dose of 100 Gy gave a similar level of fertility (U = 3.0, P = 0.51, Fig 2A) and viability of eggs (U = 6.0, P = 0.51, Fig 2B) as in the controls. Irradiation with 500 Gy or 1000 Gy reduced animal fertility significantly (U = 9.0, p = 0.046 for both cases) to < 5% and no laid eggs hatched. Finally, doses above 1000 Gy induced complete infertility.

### Effects of irradiation on eggs at different stages of development

**Fig 3:**
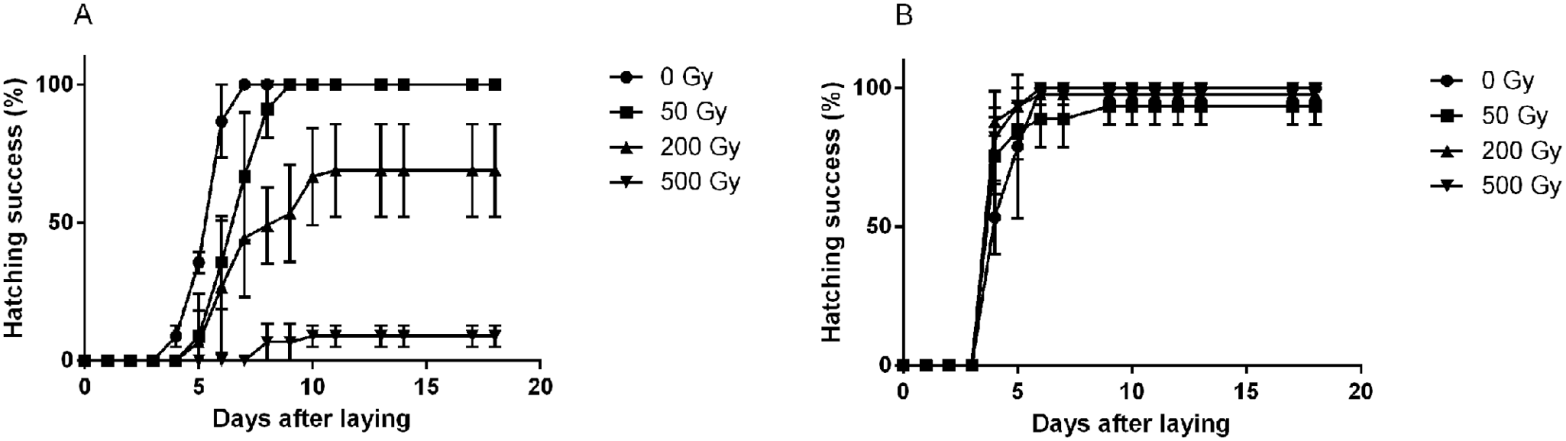
Hatching sequence and success of *H*. *dujardini* eggs irradiated at different doses and at two different stages of egg development. (A) Irradiation at the early developmental stage. (B) Irradiation at the late developmental stage. Time in days after laying are given at the x-axis. Error bars represent standard deviations from 3 repeats, each with 15 eggs.

The effects of irradiation on hatchability of eggs in the early and late developmental stages are shown in Fig 3A and 3B, respectively. There was a clear dose response for hatchability of eggs irradiated at the early developmental stage, with no effect of 50 Gy (100%, SD = 0) compared to controls (100%, SD = 0), but a significant effect of 200 Gy (69%, SD = 13.7; U = 9.0, p = 0.037) and 500 Gy (9%, SD = 3.1; U = 9.0, p = 0.034), and also a tendency of a dose-dependent delay in hatching time (Fig 3A). In contrast, late-stage embryos showed high resistance to radiation, and no significant reduction of hatchability could be observed for doses up to 500 Gy (0 Gy = 100% (SD = 0), 50 Gy = 93% (SD = 5.4), 200 Gy = 98% (SD = 3.1), 500 Gy = 100% (SD = 0); Kruskal-Wallis Analysis of differences between dose groups at day 13: H = 4.7, p = 0.20; Fig 3B).

**Fig 4:**
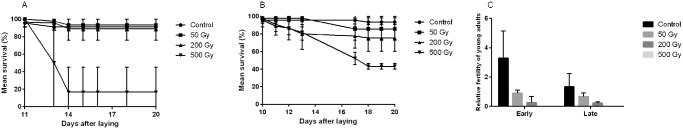
Survival of *H*. *dujardini* juveniles hatched from eggs irradiated during early or late stage of embryonic development, and their relative fertility. (A) Young adult survival after irradiation in the early stage of egg development. (B) Young adult survival after irradiation in the late stage of development. (C) Relative fertility of young adults irradiated in the early and late stages, calculated as mean number of eggs laid (in each replicate sample) divided by the highest number of young adults available per repeat.

We also analyzed the post-hatching effects in eggs that survived irradiation during the early or late stage of development. Survival of juveniles hatched from eggs irradiated at 50 Gy and 200 Gy in the early developmental stage (Fig 4A) did not differ significantly from controls (p>0.05; see S1 Text) at any measured times (survival estimate at day 20: 91.1% (SD = 3.1) for controls, 93.3% (SD = 5.4) for the 50 Gy group and 89.1% (SD = 10.4) for 200 Gy). Eggs irradiated at 500 Gy had significantly reduced survival (p<0.05) from day 14 onwards (survival estimate at day 20: 16.6% (SD = 23.5) for the 500 Gy group. Note however that only 1–2 eggs hatched in the 500 Gy group). The survival of animals from eggs irradiated at 50 Gy and 200 Gy in the late developmental stage did not differ significantly from controls (p>0.10) at any time (survival estimate at day 20: 93.3% (SD = 5.4) for controls, 85.6% (SD = 10.2) for the 50 Gy group, 75.4% (SD = 12.3) for the 200 Gy group). Also the 500 Gy group did not differ from the controls up to day 13, but survival then declined significantly from day 17 onwards (p≤0.05), and reached 43.2% (SD = 2.7) on day 20.

The relative fertility (eggs/individual) of the young adults is shown in Fig 4C. For both the early and late stage irradiation category there was a significant overall difference in relative fertility among dose groups (Early: H = 9.3, p = 0.025; Late: H = 10.1, p = 0.018). In the early stage irradiation category mean relative fertility of controls was considerably higher (3.28 eggs/ind., SD = 1.52) than in the irradiated groups (50 Gy: 0.91 eggs/ind., SD = 0.16; 200 Gy: 0.25 eggs/ind., SD = 0.36), but due to high variation within the control group only the 200 and 500 Gy groups reached statistical significance (control vs. 50 Gy: p = 0.077; control vs. 200 Gy: U = 9.0, p = 0.046; control vs. 500 Gy: U = 9.0, p = 0.037). For the late stage irradiation group there was no significant difference in relative fertility between controls (1.3 eggs/ind., SD = 0.75) and the 50 Gy group (0.7 eggs/ind., SD = 0.19, U = 8.0, p = 0.13), but the higher dose groups had marginally significantly (200 Gy: 0.2 eggs/ind., SD = 0.07, U = 9.0, p = 0.05) or significantly lower relative fertility (500 Gy: no eggs, U = 9.0, p = 0.037). The 500 Gy group differed significantly also from the 50 Gy and 200 Gy groups (U = 9.0, p = 0.037 for both cases).

The additional eggs irradiated with 50 Gy in the early stage of development and its respective controls were photographed and analyzed with respect to the description of egg developmental stages provided by Gabriel et al. [14]. S1 Fig shows the effect of gamma irradiation on cells in the initial stage of development as an apparent loss of cells 0.5h after the treatment. In the control eggs cell proliferation is evident and the developmental stages are similar in timing to the ones described by Gabriel et al. [14]. During the following days, the irradiated eggs continued their development through almost all the stages but with some time delay compared to the controls.

### Effects of low temperature during irradiation on survival and fertility of adults

**Fig 5:**
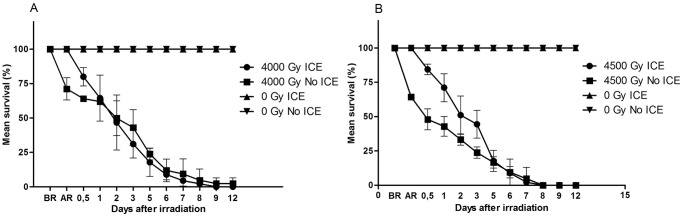
Effects of low temperature at irradiation on adult *H*. *dujardini* survival. The figure shows estimates of mean survival over time after irradiation up to 12 days post-irradiation, for samples irradiated on ice (low temperature) and without ice (20°C), and for controls kept at 20°C. Fig (A) shows data for irradiation with 4500 Gy, and Fig (B) for irradiation with 4000 Gy. BR represents survival before radiation and AR represents survival immediately after irradiation. Each data point represents the mean of three repeats, each with 15 animals.

We analyzed if the temperature at which adult tardigrades were irradiated influenced survival for doses of 4000 and 4500 Gy. At low temperature survival rate was high (100%) when measured immediately after irradiation, while irradiation at room temperature (no ice) reduced survival significantly, to 71% in the 4000 Gy group and to 64% in the 4500 Gy group (p<0.05 in both cases; Fig 5A and 5B; S1 Text). After this initial difference in survival there were only marginal or no differences in survival between the temperature regimes for 4000 Gy (p≥0.05; Fig 5A), while for the 4500 Gy group the room temperature group had lower survival up to 3 days after irradiation (p<0.05; Fig 5B), but did not differ thereafter.

**Fig 6:**
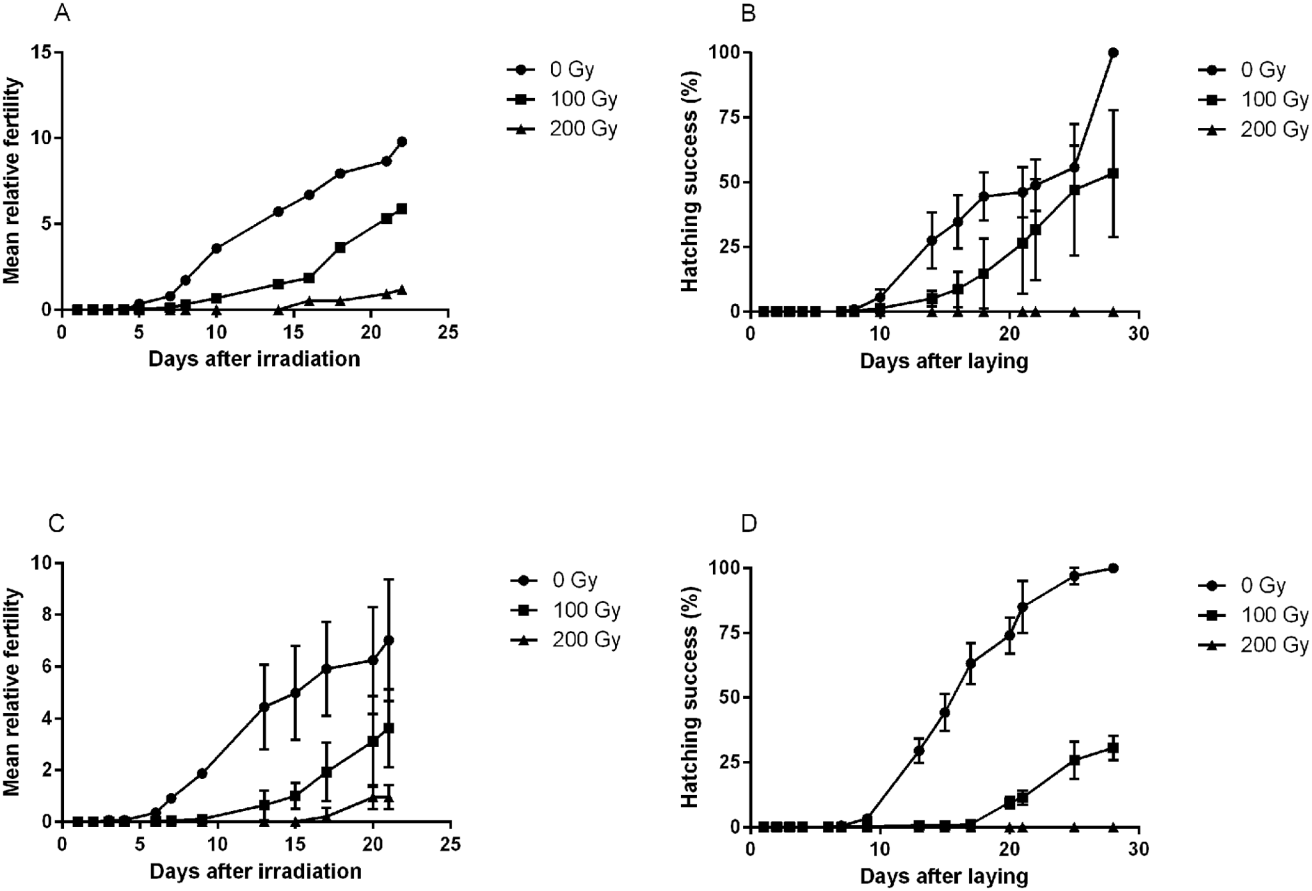
Mean relative fertility of adult *H*. *dujardini* and hatching success of laid eggs after irradiation on ice (A, B) or at room temperature (C, D). Fertility data points represent the mean number of eggs laid per individual within each repeat sample. Three repeats were used for each dose. Hatching success represents the proportion of laid eggs that hatched within each repeat sample.

We also evaluated if irradiation temperature had an effect on fertility and viability on eggs for dose levels of 0, 100 and 200 Gy (Fig 6). As shown above, irradiation > 1000 Gy resulted in complete infertility. The general dose-response in relative fertility (eggs/individuals) was very similar for samples irradiated on ice and at room temperature (Fig 6A and 6C), and there was no statistical difference in the production of eggs (pair-wise comparisons between ice and room temperature for each dose-group, p>0.05 in all analyses). There was a clear and significant dose-response both in total relative fertility (lower fertility at higher doses, H = 13.7, p = 0.001) and in the temporal patterns of egg laying (more delay at higher doses). Tardigrades irradiated at 200 Gy started to produce eggs only after 15 days, but the eggs laid were not viable (Fig 6B and 6D). Also in hatching success there was a significant dose-dependent difference among the three dose groups (H = 16.3, p<0.001) and an apparent dose-dependent delay in hatching (Fig 6B and 6D). For eggs irradiated at 100 Gy, those kept on ice tended to hatch slightly faster than those kept at room temperature (Fig 6B and 6D).

## Discussion

The present study adds a freshwater species, *H*. *dujardini*, to the list of tardigrades showing a very high tolerance to gamma radiation in the active hydrated state, with an LD50_48h_ of ~4500 Gy. This is in the same range of tolerance as previously reported for the limno-terrestrial tardigrades *Milnesium tardigradum* (5000 Gy [4]) and *Richtersius coronifer* (3500 Gy/22 hours; [3]). Since radiation tolerance is hypothesized to be a by-product of evolved mechanisms for desiccation tolerance [12], the high tolerance of *H*. *dujardini* is unexpected although our knowledge on desiccation tolerance in *H*. *dujardini* is rather limited. However, Wright [13] reported that this species was less tolerant to desiccation at low relative humidity (RH) compared to several other species (including *M*. *tardigradum*), and that successful anhydrobiosis in this species required pre-desiccation under high humidity conditions (85% RH). Thus, our results provide an interesting input to the question of the origin of tolerance to radiation in tardigrades, and the extent to which tolerances to radiation and desiccation are based on the same mechanisms.

The high tolerance gamma radiation in *H*. *dujardini* also contrasts with previous findings [7] that this species has much lower tolerance to UV radiation than the limno-terrestrial species *H*. *varieornatus*. Also other studies have documented differences among tardigrade species in tolerance to UV radiation. Dehydrated *Milnesium tardigradum* survived exposure to UV radiation in space much better than *R*. *coronifer* [8], despite similar tolerance to gamma radiation. Thus, there seems to be more differences among tardigrade species in the sensitivity to UV radiation than to ionizing radiation. The molecular mechanisms behind this pattern is unclear, but can relate to, e.g., mechanisms of DNA repair, antioxidant protection or shielding by pigmentation. The impacts of gamma radiation and UV light on DNA are generally different (double strand breaks and tymidine dimers, respectively) and also the repair pathways. However, both types of radiation induce reactive oxigen species (ROS) [15, 16]. In the study by Horikawa et al. [7] the induction of thymidine dimers by UV-C was similar in *H*. *dujardini* and *H*. *varieornatus*, confirming similar effects of radiation on DNA in these species despite their different tolerance to UV. In the latter species, the photolyase gene phrA (connected with DNA repair) was strongly upregulated in UV-C irradiated animals, while in *H*. *dujardini* no homologous phrA gene was found based on the available EST library for this species. Therefore, possibly the sensitivity of *H*. *dujardini* to UV-C could be associated with an evolutionary loss or mutation of the phrA gene/protein involved, as it seems has happened in humans [16], but the high tolerance to ionizing radiation remains to be explained.

In contrast to the high radiation tolerance in adult *H*. *dujardini*, both fertility and hatchability of eggs were affected by much lower doses of radiation. Doses above 100 Gy resulted in reduced fertility and viability of eggs. Similar results have been reported in *M*. *tardigradum*, where irradiation of juveniles with high doses (1000 Gy or higher) of gamma rays made them sterile [4]. These effects are clearly expected, since embryonic tissues are known to be more radio-sensitive than somatic tissues due to higher level of cell proliferation, and the fact that DNA damage from radiation is to a large extent expressed in proliferating cells [17,18,19]. This difference in radio-sensitivity may be particularly pronounced in tardigrades since this animal group is known to be largely (but not strictly) eutelic, i.e having a constant cell number and little somatic mitosis in the adult stage [20]. The effect on fertility in our study is therefore likely to be a direct result of higher cell division in the germinal cells due to meiosis and mitosis processes. Additional support for this conclusion come from the fact that irradiation of *H*. *dujardini* eggs at the first stage of development (well within the period of rapid cell division, as documented by Gabriel et al. [14]) led to considerable reduction in hatchability, while irradiation in the late stage (low mitotic activity) had no effect. Similar results were obtained in our previous studies in *M*. *tardigradum* [11] and *R*. *coronifer* [10]. However, there seems to be some interspecific differences with respect to sensitivity at different developmental stages. Eggs of *H*. *dujardini* in the initial stage were more tolerant than *M*. *tardigradum* eggs, since in the latter species hatchability was reduced to around 75% with 50 Gy [11], while *H*. *dujardini* eggs reached 100% hatchability at the same dose.

The irradiation of embryonic cells (eggs) in our study also revealed two additional effects. First, there was a delay in time of hatching. Second, some dividing cells were apparently lost after irradiation exposure (see S1 Fig). Most metazoan embryos show fast cell division cycles at the beginning of their development [21], and DNA damage during cell divisions usually triggers cell cycle arrest providing time for repair or apoptosis induction [22,23]. However, in the early developmental stage of *C*. *elegans*, *D*. *melanogaster* and *X*. *laevis* embryos, checkpoint activation to stop the cell cycle after DNA damage is highly or slightly silenced during the fast cell cycle divisions, and activated at the end of midblastula transition [24]. In a cell ablation experiment with tardigrade embryos using a laser beam, Hejnol [25] removed half of the 4-cell embryo in *Thulinia stephaniae*. Remarkably, the development proceeded normally and the eggs eventually hatched, but with a time delay compared to the controls. Such developmental delays were also observed in the present study, and in previous radiation studies in *M*. *tardigradum* [11], indicating that the early embryogenesis is highly regulated in tardigrades [25]. After the apparent cell loss caused by irradiation in our study, the rest of the cells in the *H*. *dujardini* embryo were able to continue development but were delayed compared to the control.

In many animals genome integrity is subject to stringent DNA damage detection, repair, or induction of apoptosis in cells with low genomic fidelity [26]. Indications of apoptosis avoidance have been shown in mice oocytes (early stage, meiotic) lacking specific apoptosis regulator genes (*Puma* and *Noxa*). The absence of these genes protected them from γ-irradiation-induced apoptosis and generated healthy offspring [26]. In the desiccation and radiation tolerant chironomid *Polypedilum vanderplanki*, radioresistance was associated with repair of damaged DNA, and repair was also indicated after desiccation [27]. However, in *P*. *vanderplanki* the process of repair is apparently rather slow (24h after 70 Gy gamma rays, and 168h after heavy ions ^4^He), and during this repair period apoptosis is prevented. The apparent recovery in fertility observed in our study 15 days after adult irradiation with 200 Gy might also be an indication of a similar slow repair process in tardigrades.

Even though the irradiated but surviving embryonic cells were able to produce an embryo capable of hatching and developing into young adults, there was an apparent reduction in their fertility as the dose increased. This indicates that surviving cells were still affected in later stages of the life cycle. Moreover, the strong decline in survival of young adults originating from eggs irradiated with 500 Gy during the late stage of egg development is also an indication of residual damage, affecting life span and fertility. Studies on rotifers and *C*. *elegans* have shown that radiation does not affect fertility in terms of egg number, but rather induces sterility [28]. According to these studies, the cells in the eggs were not directly killed by radiation, but received damage that was expressed later in life.

The role of an efficient DNA repair system has previously been suggested as a possible explanation for the high tolerance to radiation in tardigrades [11,12,29,30,31], but has yet to be documented. However, two important proteins for DNA damage/response are induced in tardigrades after radiation exposure (RAD51, [31]; HSP70, [32]). Also, in response to desiccation, the polyubiquitin gene (UBB) related to the regulation of DNA repair mechanisms has been reported to be up-regulated in *M*. *tardigradum* [33]. Protein protection may also contribute to radiation tolerance, and Krisko et al. [28] reported a negative correlation between fecundity and protein carbonylation in the rotifer *A*. *vaga* and the nematode *C*. *elegans* after radiation exposure. Apparently, the antioxidant protection, in terms of less protein carbonylation, is higher in *A*. *vaga* which is more resistant to radiation compared to *C*. *elegans*. Thus, the concept of protein protection, including DNA repair proteins, should be evaluated as a possible mechanism of radiation tolerance in multicellular organisms [28].

Irradiation of tardigrades at different temperatures did not influence survival rates but low temperature resulted in a delay by 12 to 24 hours in the effect of gamma radiation. Irradiation on ice is a common procedure in many experiments of mammalian cells in order to reduce activation of DNA repair, along with other cellular processes, during the radiation exposure [34,35]. Recent studies have also shown that dehydration and low temperatures (below freezing) can provide protection to radiation damage by reducing the diffusion of free radicals [36–38]. It is well known in radiation biology that 70% of the DNA damage is caused by free radicals (Low LET) in hydrated conditions. According to Slade and Radman [39], the survival rate of *D*. *radiodurans* is higher when the irradiation is done on dry ice (-70°C) compared to room temperature or ice [36], and they suggested that this resistance rely on diminished reactive oxygen effects on proteins under frozen irradiation conditions. However, in the present study there was no evidence of a protective effect of low temperature (0°C), and the delay in mortality is not likely to be explained by effects of radical chemistry. A more suitable explanation might be a biological response caused by the high level of DNA damage, which may have triggered the animals’ death after the temperature rose.

In summary, our study shows that fresh water tardigrades are also able to tolerate high doses of gamma radiation, with similar patterns of dose-response as limno-terrestrial tardigrades. Assuming that adult tardigrades and late developmental stages of embryos have only a few cells mitotically active (due to eutely), we suggest that this might explain part of the observed radiation tolerance. However, efficient DNA repair mechanisms may still contribute to the high radiation tolerance observed, both in adult tardigrades and in embryos relative to other animals. Further studies are needed to evaluate the relative importance of eutely vs. other mechanisms (e.g., DNA repair system, protein protection, apoptosis avoidance) in radiation tolerance of tardigrades.

## Supporting Information

S1 Fig Gamma radiation effect on eggs at the early developmental stage. The figure show images of 2 representative irradiated eggs (IR, 50 Gy) and 2 control eggs at different times in the development. Start measurement picture (before irradiation) was taken after release of eggs into the exuvia. Pictures were then taken immediately after the irradiation, and at different time intervals up to 137 hours after irradiation. The corresponding developmental time and cell/embryo morphology stages is given in the figure. See Methods section for more details on these stages. Bar scale 10 μm.
(TIF)

S1 Text Statistical results related to figures in the article. (DOCX)
