# Supplementary figures and images for: Tolerance to Gamma Radiation in the Tardigrade Hypsibius dujardini from Embryo to Adult Correlate Inversely with Cellular Proliferation
Source: PLoS One. 2015 Jul 24;10(7):e0133658. doi: 10.1371/journal.pone.0133658 (PMC4514856; doi:10.1371/journal.pone.0133658)

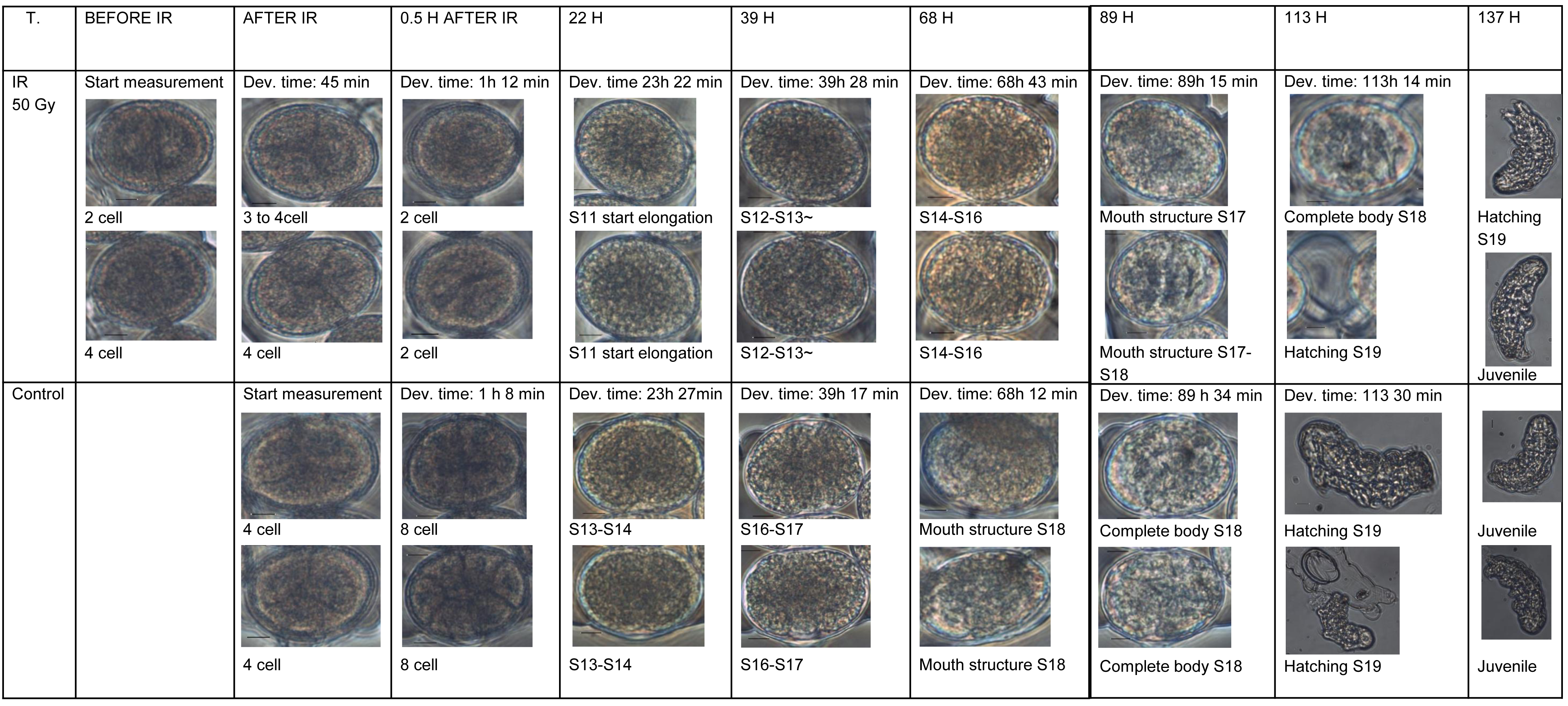

Supplement: S1 Fig — The figure show images of 2 representative irradiated eggs (IR, 50 Gy) and 2 control eggs at different times in the development. Start measurement picture (before irradiation) was taken after release of eggs into the exuvia. Pictures were then taken immediately after the irradiation, and at different time intervals up to 137 hours after irradiation. The corresponding developmental time and cell/embryo morphology stages is given in the figure. See Methods section for more details on these stages. Bar scale 10 μm. (TIF) [file pone.0133658.s001.tif]
