# Supplementary material for: Tolerance to Gamma Radiation in the Tardigrade Hypsibius dujardini from Embryo to Adult Correlate Inversely with Cellular Proliferation
Source: PLoS One. 2015 Jul 24;10(7):e0133658. doi: 10.1371/journal.pone.0133658 (PMC4514856; doi:10.1371/journal.pone.0133658)
Supplement: S1 Text — (DOCX) [file pone.0133658.s002.docx]

**Supporting Information File 2**

Statistical results related to different figures in the article.

**Figure 1.**

Overall analysis including data for dose groups 0 to 5000 Gy:

Day 1 post-irradiation: H=31.97, p=0.000, df=10.

Day 2 post-irradiation: H=28.73, p=0.001, df=10.

Day 3 post-irradiation: H=27.38, p=0.002, df=10.

Day 5 post-irradiation: H=27.77, p=0.002, df=10.

Day 7 post-irradiation: H=26.64, p=0.003, df=10.

Day 9 post-irradiation: H=26.97, p=0.003, df=10.

Day 11 post-irradiation: H=27.49, p=0.002, df=10.

Day 12 post-irradiation: H=27.55, p=0.002, df=10.

Overall analysis including data for dose groups 0 to 3000 Gy:

Day 1 post-irradiation: H=0.00, p=1.00, df=7.

Day 2 post-irradiation: H=6.27, p=0.51, df=7.

Day 3 post-irradiation: H=5.48, p=0.60, df=7.

Day 5 post-irradiation: H=5.48, p=0.60, df=7.

Day 7 post-irradiation: H=4.60, p=0.71, df=7.

Day 9 post-irradiation: H=7.96, p=0.34, df=7.

Day 11 post-irradiation: H=13.47, p=0.062, df=7.

Day 12 post-irradiation: H=13.65, p=0.058, df=7.

Since no significant overall differences were found, no pairwise comparisons were made.

Pairwise comparisons of control, 4000 Gy, 4500 Gy and 5000 Gy:

*Day 1 post-irradiation:* 0 vs. 4000 Gy, U=9.00, p=0.037, df=1.

0 vs. 4500 Gy, U=9.00, p=0.025, df=1.

0 vs. 5000 Gy, U=9.00, p=0.025, df=1.

4000 vs. 4500 Gy, U=9.00, p=0.037, df=1.

4000 vs. 5000 Gy, U=9.00, p=0.037, df=1.

4500 vs. 5000 Gy, U=9.00, p=0.025, df=1.

*Day 2 post-irradiation:* 0 vs. 4000 Gy, U=9.00, p=0.037, df=1.

0 vs. 4500 Gy, U=9.00, p=0.037, df=1.

0 vs. 5000 Gy, U=9.00, p=0.025, df=1.

4000 vs. 4500 Gy, U=9.00, p=0.050, df=1.

4000 vs. 5000 Gy, U=9.00, p=0.037, df=1.

4500 vs. 5000 Gy, U=9.00, p=0.037, df=1.

*Day 3 post-irradiation:* 0 vs. 4000 Gy, U=9.00, p=0.034, df=1.

0 vs. 4500 Gy, U=9.00, p=0.034, df=1.

0 vs. 5000 Gy, U=9.00, p=0.025, df=1.

4000 vs. 4500 Gy, U=9.00, p=0.043, df=1.

4000 vs. 5000 Gy, U=9.00, p=0.034, df=1.

4500 vs. 5000 Gy, U=9.00, p=0.034, df=1.

*Day 5 post-irradiation:* 0 vs. 4000 Gy, U=9.00, p=0.037, df=1.

0 vs. 4500 Gy, U=9.00, p=0.034, df=1.

0 vs. 5000 Gy, U=9.00, p=0.037, df=1.

4000 vs. 4500 Gy, U=9.00, p=0.046, df=1.

4000 vs. 5000 Gy, U=9.00, p=0.050, df=1.

4500 vs. 5000 Gy, U=9.00, p=0.046, df=1.

*Day 7 post-irradiation:* 0 vs. 4000 Gy, U=9.00, p=0.046, df=1.

0 vs. 4500 Gy, U=9.00, p=0.043, df=1.

0 vs. 5000 Gy, U=9.00, p=0.043, df=1.

4000 vs. 4500 Gy, U=9.00, p=0.046, df=1.

4000 vs. 5000 Gy, U=9.00, p=0.046, df=1.

4500 vs. 5000 Gy, U=9.00, p=0.043, df=1.

*Day 9 post-irradiation:* 0 vs. 4000 Gy, U=9.00, p=0.046, df=1.

0 vs. 4500 Gy, U=9.00, p=0.043, df=1.

0 vs. 5000 Gy, U=9.00, p=0.034, df=1.

4000 vs. 4500 Gy, U=9.00, p=0.046, df=1.

4000 vs. 5000 Gy, U=9.00, p=0.037, df=1.

4500 vs. 5000 Gy, U=9.00, p=0.034, df=1.

*Day 11 post-irradiation:* 0 vs. 4000 Gy, U=9.00, p=0.034, df=1.

0 vs. 4500 Gy, U=9.00, p=0.034, df=1.

0 vs. 5000 Gy, U=9.00, p=0.025, df=1.

4000 vs. 4500 Gy, U=6.00, p=0.50, df=1.

4000 vs. 5000 Gy, U=9.00, p=0.034, df=1.

4500 vs. 5000 Gy, U=6.00, p=0.32, df=1.

*Day 12 post-irradiation:* 0 vs. 4000 Gy, U=9.00, p=0.034, df=1.

0 vs. 4500 Gy, U=9.00, p=0.025, df=1.

0 vs. 5000 Gy, U=9.00, p=0.025, df=1.

4000 vs. 4500 Gy, U=9.00, p=0.034, df=1.

4000 vs. 5000 Gy, U=9.00, p=0.034, df=1.

4500 vs. 5000 Gy, U=4.50, p=1.00, df=1.

**Figure 2A**

0 vs. 100 Gy, U=3.00, p=0.51, df=1.

0 vs. 500 Gy, U=9.00, p=0.046, df=1.

0 vs. 1000 Gy, U=9.00, p=0.046, df=1.

100 vs. 500 Gy, U=9.00, p=0.046, df=1.

100 vs. 1000 Gy, U=9.00, p=0.046, df=1.

500 vs. 1000 Gy, U=5.00, p=0.81, df=1.

**Figure 2B**

0 vs. 100 Gy, U=6.00, p=0.51, df=1.

0 vs. 500 Gy, U=9.00, p=0.037, df=1.

0 vs. 1000 Gy, U=9.00, p=0.037, df=1.

100 vs. 500 Gy, U=9.00, p=0.037, df=1.

100 vs. 1000 Gy, U=9.00, p=0.037, df=1.

500 vs. 1000 Gy, U=4.50, p=1.00, df=1.

**Figure 3**

Relevant statistics reported in the main text.

**Figure 4A**

*Day 11 post-hatch*

0 vs. 50 Gy; U=1.5, p=0.11.

0 vs. 200 Gy; U=6.5, p=0.37.

0 vs. 500 Gy; U=1.5, p=0.11.

0 vs. 50 Gy; U=7.5, p=0.12.

50 vs. 500 Gy; U=4.5, p=1.00.

200 vs. 500 Gy; U=1.5, p=0.12.

*Day 13 post-hatch*

0 vs. 50 Gy; U=1.0, p=0.099.

0 vs. 200 Gy; U=5.0, p=0.83.

0 vs. 500 Gy; U=6.0, p=0.51.

0 vs. 50 Gy; U=7.0, p=0.25.

50 vs. 500 Gy; U=7.0, p=0.25.

200 vs. 500 Gy; U=6.5, p=0.38.

*Day 14 post-hatch*

0 vs. 50 Gy; U=3.5, p=0.64.

0 vs. 200 Gy; U=6.0, p=0.51.

0 vs. 500 Gy; U=9.0, p=0.043.

0 vs. 50 Gy; U=6.5, p=0.38.

50 vs. 500 Gy; U=9.0, p=0.046.

200 vs. 500 Gy; U=9.0, p=0.046.

*Day 15 post-hatch*

0 vs. 50 Gy; U=3.5, p=0.64.

0 vs. 200 Gy; U=6.0, p=0.51.

0 vs. 500 Gy; U=9.0, p=0.043.

0 vs. 50 Gy; U=6.5, p=0.38.

50 vs. 500 Gy; U=9.0, p=0.046.

200 vs. 500 Gy; U=9.0, p=0.046.

*Day 16 post-hatch*

0 vs. 50 Gy; U=3.5, p=0.64.

0 vs. 200 Gy; U=6.0, p=0.51.

0 vs. 500 Gy; U=9.0, p=0.043.

0 vs. 50 Gy; U=6.5, p=0.38.

50 vs. 500 Gy; U=9.0, p=0.046.

200 vs. 500 Gy; U=9.0, p=0.046.

*Day 18 post-hatch*

0 vs. 50 Gy; U=3.5, p=0.64.

0 vs. 200 Gy; U=6.0, p=0.51.

0 vs. 500 Gy; U=9.0, p=0.043.

0 vs. 50 Gy; U=6.5, p=0.38.

50 vs. 500 Gy; U=9.0, p=0.046.

200 vs. 500 Gy; U=9.0, p=0.046.

*Day 20 post-hatch*

0 vs. 50 Gy; U=4.0, p=0.82.

0 vs. 200 Gy; U=6.0, p=0.51.

0 vs. 500 Gy; U=9.0, p=0.043.

0 vs. 50 Gy; U=6.5, p=0.38.

50 vs. 500 Gy; U=9.0, p=0.046.

200 vs. 500 Gy; U=9.0, p=0.046.

**Figure 4B**

*Day 10 post-hatch*

0 vs. 50 Gy; U=4.0, p=0.80.

0 vs. 200 Gy; U=5.0, p=0.81.

0 vs. 500 Gy; U=4.0, p=0.80.

0 vs. 50 Gy; U=7.0, p=0.24.

50 vs. 500 Gy; U=5.0, p=0.80.

200 vs. 500 Gy; U=4.0, p=0.81.

*Day 11 post-hatch*

0 vs. 50 Gy; U=4.0, p=0.80.

0 vs. 200 Gy; U=6.5, p=0.35.

0 vs. 500 Gy; U=6.5, p=0.35.

0 vs. 50 Gy; U=7.0, p=0.25.

50 vs. 500 Gy; U=7.0, p=0.25.

200 vs. 500 Gy; U=4.0, p=0.82.

*Day 12 post-hatch*

0 vs. 50 Gy; U=4.0, p=0.80.

0 vs. 200 Gy; U=6.5, p=0.35.

0 vs. 500 Gy; U=7.5, p=0.18.

0 vs. 50 Gy; U=7.0, p=0.25.

50 vs. 500 Gy; U=8.5, p=0.072.

200 vs. 500 Gy; U=4.5, p=1.00.

*Day 13 post-hatch*

0 vs. 50 Gy; U=4.0, p=0.80.

0 vs. 200 Gy; U=7.0, p=0.25.

0 vs. 500 Gy; U=8.0, p=0.099.

0 vs. 50 Gy; U=7.0, p=0.25.

50 vs. 500 Gy; U=9.0, p=0.043.

200 vs. 500 Gy; U=4.0, p=0.83.

*Day 17 post-hatch*

0 vs. 50 Gy; U=7.0, p=0.25.

0 vs. 200 Gy; U=8.0, p=0.12.

0 vs. 500 Gy; U=9.0, p=0.046.

0 vs. 50 Gy; U=7.0, p=0.28.

50 vs. 500 Gy; U=9.0, p=0.050.

200 vs. 500 Gy; U=9.0, p=0.050.

*Day 18 post-hatch*

0 vs. 50 Gy; U=6.5, p=0.38.

0 vs. 200 Gy; U=8.0, p=0.12.

0 vs. 500 Gy; U=9.0, p=0.050.

0 vs. 50 Gy; U=7.0, p=0.27.

50 vs. 500 Gy; U=9.0, p=0.050.

200 vs. 500 Gy; U=9.0, p=0.046.

*Day 19 post-hatch*

0 vs. 50 Gy; U=6.5, p=0.38.

0 vs. 200 Gy; U=8.0, p=0.12.

0 vs. 500 Gy; U=9.0, p=0.050.

0 vs. 50 Gy; U=7.0, p=0.27.

50 vs. 500 Gy; U=9.0, p=0.050.

200 vs. 500 Gy; U=9.0, p=0.046.

**Figure 5A,B.**

Results for 4000 and 4500 Gy refer to figure 5A and 5B, respectively.

*Directly after irradiation:*

4000 Gy ice vs. no ice; U=9.00, p=0.037.

4500 Gy ice vs. no ice; U=9.00, p=0.025.

*12h post-irradiation:*

4000 Gy ice vs. no ice; U=9.00, p=0.050.

4500 Gy ice vs. no ice; U=9.00, p=0.046.

*1 day post-irradiation:*

4000 Gy ice vs. no ice; U=6.00, p=0.51.

4500 Gy ice vs. no ice; U=9.00, p=0.050.

*2 days post-irradiation:*

4000 Gy ice vs. no ice; U=4.00, p=0.83.

4500 Gy ice vs. no ice; U=9.00, p=0.046.

*3 days post-irradiation:*

4000 Gy ice vs. no ice; U=2.00, p=0.28.

4500 Gy ice vs. no ice; U=9.00, p=0.046.

*5 days post-irradiation:*

4000 Gy ice vs. no ice; U=2.50, p=0.38.

4500 Gy ice vs. no ice; U=3.00, p=0.50.

*6 days post-irradiation:*

4000 Gy ice vs. no ice; U=4.00, p=0.83.

4500 Gy ice vs. no ice; U=3.00, p=0.51.

*7 days post-irradiation:*

4000 Gy ice vs. no ice; U=4.50, p=1.00.

4500 Gy ice vs. no ice; U=4.00, p=0.80.

*8 days post-irradiation:*

4000 Gy ice vs. no ice; U=4.00, p=0.80.

4500 Gy ice vs. no ice; U=4.50, p=1.00.

*9 days post-irradiation:*

4000 Gy ice vs. no ice; U=3.00, p=0.32.

4500 Gy ice vs. no ice; U=4.50, p=1.00.

*12 days post-irradiation:*

4000 Gy ice vs. no ice; U=3.00, p=0.32.

4500 Gy ice vs. no ice; U=4.50, p=1.00.
